# Tricuspid regurgitation after cardiac resynchronization therapy: evolution and prognostic significance^[Author-notes euac034-FM1]^

**DOI:** 10.1093/europace/euac034

**Published:** 2022-03-28

**Authors:** Jan Stassen, Xavier Galloo, Kensuke Hirasawa, Nina Ajmone Marsan, Pieter van der Bijl, Victoria Delgado, Jeroen J Bax

**Affiliations:** Department of Cardiology, Leiden University Medical Center, Albinusdreef 2, 2300 RC Leiden, The Netherlands; Department of Cardiology, Jessa Hospital, Stadsomvaart 11, 3500 Hasselt, Belgium; Department of Cardiology, Leiden University Medical Center, Albinusdreef 2, 2300 RC Leiden, The Netherlands; Department of Cardiology, Universitair Ziekenhuis Brussel, Laarbeeklaan 101, 1090 Brussels, Belgium; Department of Cardiology, Leiden University Medical Center, Albinusdreef 2, 2300 RC Leiden, The Netherlands; Department of Cardiology, Leiden University Medical Center, Albinusdreef 2, 2300 RC Leiden, The Netherlands; Department of Cardiology, Leiden University Medical Center, Albinusdreef 2, 2300 RC Leiden, The Netherlands; Department of Cardiology, Leiden University Medical Center, Albinusdreef 2, 2300 RC Leiden, The Netherlands; Department of Cardiology, Leiden University Medical Center, Albinusdreef 2, 2300 RC Leiden, The Netherlands; Department of Cardiology, Turku Heart Center, University of Turku and Turku University Hospital, Kiinamyllynkatu 4-8, FI-20520 Turku, Finland

**Keywords:** Cardiac resynchronization therapy, Tricuspid regurgitation, Heart failure, Mortality, Outcome, Prognosis

## Abstract

**Aims:**

Tricuspid regurgitation (TR) is common in patients with heart failure (HF) and is associated with worse outcome. This study investigated the effect of cardiac resynchronization therapy (CRT) on TR severity and long-term outcome.

**Methods and results:**

Tricuspid regurgitation severity was assessed at baseline and 6 months after CRT implantation, using a multiparametric approach. Patients were divided into four groups: (i) no or mild TR without progression; (ii) no or mild TR with progression to significant (moderate–severe) TR; (iii) significant TR with improvement to no or mild TR; and (iv) significant TR without improvement. The primary endpoint was all-cause mortality. A total of 852 patients (mean age 65 ± 11 years, 77% male) were included. At baseline, 184 (22%) patients had significant TR, with 75 (41%) showing significant improvement at 6-month follow-up. After a median follow-up of 92 (50–137) months, 494 (58%) patients died. Patients with significant TR showing improvement at follow-up had better outcomes than those showing no improvement (*P* = 0.016). On multivariable analysis, no or mild TR progressing to significant TR [hazard ratio (HR) 1.745; 95% confidence interval (CI): 1.287–2.366; *P* < 0.001] and significant TR without improvement (HR 1.572; 95% CI: 1.198–2.063; *P* = 0.001) were independently associated with all-cause mortality, whereas significant TR with improvement at follow-up was not (HR: 1.153; 95% CI: 0.814–1.633; *P* = 0.424).

**Conclusion:**

Improvement of significant TR after CRT is observed in a substantial proportion of patients, highlighting the potential benefit of CRT for patients with HF having significant TR. Significant TR at 6 months after CRT is independently associated with increased long-term mortality.

**What’s new?**

- Moderate–severe tricuspid regurgitation (TR) is observed in 22% of cardiac resynchronization therapy (CRT) recipients.
- Moderate–severe TR improves to none-mild TR in 41% of patients, 6 months after CRT implantation.
- Patients with TR improvement show more pronounced right ventricular reverse remodelling.
- TR improvement is associated with better functional outcome.
- TR improvement is independently associated with better survival outcome.

## Introduction

Significant (≥moderate) tricuspid regurgitation (TR) is common in patients with heart failure (HF) and reduced left ventricular ejection fraction (LVEF), with a prevalence up to 23%.^1^ In these patients, TR aetiology is most likely to be secondary, with tricuspid annular dilatation and increased tricuspid leaflet tethering due to right ventricular (RV) enlargement and dysfunction, which is often secondary to left-sided heart disease. Because of the expanding use of cardiac implantable electronic devices in HF, some individuals may have primary TR, where the device lead interferes with the normal function of the tricuspid valve apparatus.^2^

Contemporary epidemiological studies have shown that significant TR is independently associated with poor long-term prognosis.^1^ Although medical therapy can improve symptoms, it has not been demonstrated to improve outcomes in these patients.^3^ Current guidelines recommend proceeding to tricuspid valve surgery in patients undergoing left-sided valve surgery with: (i) severe functional TR or (ii) progressive functional TR and annular dilatation or symptoms/signs of right-sided HF. Surgical tricuspid valve intervention is also recommended for individuals with signs of right-sided HF and severe, isolated, functional TR who have responded suboptimally to medical therapy.^3^ However, recommendations for surgical tricuspid valve intervention are based on non-randomized data.^3^ In addition, mortality rates for tricuspid valve surgery remain high, with an overall in-hospital mortality of up to 10%.^4^ This emphasizes the need for lower risk strategies to reduce TR severity in patients with HF.^5^

Cardiac resynchronization therapy (CRT) is a well-established treatment for appropriately selected patients with HF and reduced LVEF. In addition, reduction of functional mitral regurgitation severity has been reported after CRT, related to acute resynchronization of LV papillary muscle contraction, followed by LV reverse remodelling at a later stage.^6,^^7^ Reverse remodelling of the RV has also been documented after CRT.^8^ The impact of CRT on TR severity however, has not been evaluated. Therefore, the aims of the current study were to (i) analyse the prevalence of TR in a large cohort of CRT recipients, (ii) evaluate the relationship between the evolution of TR after 6 months of CRT and the clinical and echocardiographic response to CRT, and (iii) assess the association between changes in TR severity after CRT and long-term outcomes.

## Methods

### Patient population and clinical data collection

Heart Failure patients who remained symptomatic despite receiving guideline-direct medical therapy and who received CRT between September 2000 and September 2014 according to contemporary guidelines,^9^ were included from a registry at the Leiden University Medical Center, The Netherlands. Patients who did not have transthoracic echocardiography at baseline or 6 months after CRT implantation, in whom TR severity could not be quantified, or who underwent tricuspid valve surgery (replacement or repair) after CRT implantation were excluded from the analysis (Supplementary material online, *Figure S1*). Demographic, clinical, electrocardiographic and echocardiographic data were prospectively collected before CRT implantation in the departmental cardiology information system (EPD-vision; Leiden University Medical Center, Leiden, The Netherlands) and retrospectively analysed. An ischaemic aetiology of HF was diagnosed by the presence of significant coronary artery disease on invasive coronary angiography. Quality of life was evaluated with the Minnesota Living with Heart Failure Questionnaire and, if feasible, a 6-min walk test was performed. Renal function was quantified by estimating the glomerular filtration rate with the Modification of Diet in Renal Disease Study equation. The study complies with the Declaration of Helsinki and was approved by the Institutional Review Board. Due to the retrospective study design, the Medical Ethical Committee waived the need for written informed consent on a patient level.

### Echocardiographic data acquisition and analysis

All patients underwent transthoracic echocardiography before CRT implantation in the left lateral decubitus position with commercially available ultrasound equipment (Vivid 7 and E9, GE-Vingmed, Horten, Norway). Electrocardiogram-triggered echocardiographic data were stored digitally in a cine-loop format for offline analysis using EchoPAC version 203 (GE Medical Systems, Horten, Norway). For the purpose of this study, all original echocardiographic data were re-evaluated. Left ventricular volumes, LVEF and left atrial volumes were measured using Simpson’s biplane method.^10^ Right ventricular end-systolic area and end-diastolic area were traced in a focused RV apical view according to current recommendations.^10^ For the evaluation of RV systolic function, RV fractional area change was calculated by the following formula: fractional area change = ([RV end-diastolic area − RV end-systolic area]/RV end-diastolic area) × 100%.^10^ Tricuspid annular plane systolic excursion was measured on M-mode recordings of the lateral tricuspid annulus in an RV-focused view. Right ventricular peak systolic pressure was derived from the peak velocity of the TR jet according to the Bernoulli equation, adding the right atrial pressure (estimated by the inspiratory collapse and diameter of the inferior vena cava).^10,^^11^ The severity of mitral regurgitation and TR was graded using a multiparametric approach, as recommended by current guidelines^12,^^13^ and was graded on a 5-point scale; 0 = no, 1 = mild, 2 = moderate, 3 = moderate to severe, and 4 = severe.

### Clinical endpoints

New York Heart Association (NYHA) functional class, quality of life and 6-min walk test were evaluated at 6 months after CRT implantation and related to changes in TR severity. In addition, patients were followed up for the occurrence of all-cause mortality. Data on mortality were obtained from the departmental cardiology information system (EPD-Vision, Leiden University Medical Center, Leiden, The Netherlands), which is linked to the governmental death registry database. Follow-up data were complete for all patients.

### Statistical analysis

Continuous data are presented as mean ± standard deviation when normally distributed and as median and interquartile range when not normally distributed. Categorical data are presented as frequencies and percentages. Continuous variables were compared using the analysis of variances test with Bonferroni’s *post hoc* analysis, whereas the Kruskal–Wallis test was used to compare continuous variables that did not adhere to a normal distribution. Categorical variables were compared using the Pearson χ^2^ test. Changes in clinical and echocardiographic variables, expressed as continuous data, were evaluated using the paired samples *t*-test within each group and compared between groups using linear mixed models. Event-free survival curves were generated using the Kaplan–Meier method and differences between groups were analysed using the log-rank test. To assess the association between changes in TR severity and all-cause mortality, uni- and multivariable Cox proportional hazard models were constructed, adjusting for variables known to have an impact on prognosis. The following covariates were included in the multivariable model: age, sex, arterial hypertension, diabetes mellitus, dyslipidaemia, body mass index, ischaemic aetiology of HF, atrial fibrillation, NYHA functional class III–IV, estimated glomerular filtration rate, LV end-systolic volume at 6 months, LVEF at 6 months, and relative change in RV end-systolic area at 6 months. For both uni- and multivariable analyses, hazard ratios (HRs) with 95% confidence intervals (CIs) were calculated. A two-sided *P*-value <0.05 was considered statistically significant. Statistical analysis was performed using SPSS for Windows, version 25.0 (IBM, Armonk, NY, USA).

## Results

### Clinical and echocardiographic characteristics at baseline

A total of 852 patients (mean age 65 ± 11 years, 77% male) with TR assessment at baseline and 6 months after CRT implantation were included in the study (Supplementary material online, *Figure S1*). Baseline clinical characteristics of the overall population and according to the change in TR are shown in *Table 1*, while *Table 2* summarizes the echocardiographic data. An ischaemic aetiology was present in 501 (59%) patients. The mean baseline LVEF was 27.8 ± 8.1% and the mean QRS duration was 154 ± 35 ms at baseline. A cardiovascular implantable electronic device was present in 219 (26%) patients [103 (47%) patients with a pacemaker and 116 (53%) with an implantable cardioverter-defibrillator].

**Table 1:** Baseline clinical characteristics of the patient population

	Overall study population (*n* = 852)	Baseline Grade 0–1 TR unchanged (*n* = 583)	Baseline Grade 2–4 TR improved (*n* = 75)	Baseline Grade 0–1 TR worsened (*n* = 85)	Baseline Grade 2–4 TR unchanged (*n* = 109)	*P*-value
Age (years)	65.2 (±10.5)	64.4 (±10.4)	64.2 (±12.0)	66.9 (±8.8)	68.5 (±10.4)^*,^^†^	0.001
Male sex (%)	652 (76.5%)	456 (78.2%)	49 (65.3%)	65 (76.5%)	82 (75.2%)	0.100
Arterial hypertension (%)	406 (47.8%)	284 (48.7%)	35 (47.9%)	36 (42.4%)	51 (46.8%)	0.741
Diabetes mellitus (%)	179 (21.0%)	118 (20.2%)	22 (29.3%)	14 (16.5%)	25 (22.9%)	0.200
Dyslipidaemia (%)	358 (42.2%)	252 (43.3%)	31 (41.9%)	41 (48.2%)	34 (31.5%)	0.084
Current smoker (%)	136 (16.1%)	102 (17.7%)	13 (17.8%)	13 (15.5%)	8 (7.3%)	0.141
BMI (kg/m^2^)	26.6 (±4.3)	26.8 (±4.3)	26.4 (±4.5)	26.8 (±4.2)	25.2 (±3.6)^*^	0.006
Ischaemic aetiology (%)	501 (58.8%)	335 (57.5%)	43 (57.3%)	60 (70.6%)	63 (57.8%)	0.144
QoL score	30.6 (±19.1)	28.3 (±18.2)	37.5 (±18.5)^*^	33.5 (±20.0)	34.5 (±20.4)^*^	<0.001
6MWT (m)	342 (±118)	361 (±111)	306 (±125)^*^	318 (±110)^*^	302 (±127)^*^	<0.001
NYHA III–IV (%)	537 (64.4%)	339 (59.5%)	55 (74.3%)^*^	61 (75.3%)^*^	82 (75.2%)^*^	<0.001
Sinus rhythm (%)	622 (73.0%)	466 (79.9%)	48 (64.0%)^*^	65 (76.5%)	43 (39.4%)^*,^^†,^^#^	<0.001
QRS duration (ms)	154 (±35)	152 (±34)	157 (±38)	149 (±33)	162 (±39)	0.031
Previous CIED (%)	219 (25.7%)	121 (20.8%)	24 (32.0%)	25 (29.4%)	49 (45.0%)	<0.001
Beta-blocker (%)	645 (75.7%)	453 (77.7%)	56 (74.7%)	63 (74.1%)	73 (67.0%)	0.114
ACE-i/ARB (%)	755 (88.6%)	535 (91.8%)	63 (84.0%)	70 (82.4%)^*^	87 (79.8%)^*^	<0.001
MRA (%)	379 (44.5%)	248 (42.5%)	39 (52.0%)	42 (49.4%)	50 (45.9%)	0.317
Diuretics (%)	674 (79.1%)	443 (76.0%)	61 (81.3%)	72 (84.7%)	98 (89.9%)^*^	0.005
Statin (%)	546 (64.1%)	387 (66.4%)	42 (56.0%)	57 (67.1%)	60 (55.0%)	0.054
eGFR, (mL/min/1.73 m^2^)	68.0 (±23.7)	70.4 (±23.8)	66.3 (±21.7)	63.1 (±22.7)^*^	60.2 (±23.1)^*^	<0.001
Haemoglobin (g/dL)	13.4 (±1.6)	13.5 (±1.6)	13.5 (±1.6)	13.1 (±1.6)	12.9 (±1.6)^*,^^†^	<0.001

Values are presented as mean ± SD, median (IQR), or *n* (%).

ACE-i, angiotensin-converting enzyme inhibitor; ARB, angiotensin receptor blocker; BMI, body mass index; CIED, cardiac implantable electronic device; eGFR, estimated glomerular filtration rate; MRA, mineralocorticoid receptor antagonist; MWT, minute walking test; NYHA, New York Heart Association; QoL, quality of life.

* *P* < 0.05 vs. baseline Grade 0–1 TR unchanged.

† *P* < 0.05 vs. baseline Grade 2–4 TR improved.

# *P* < 0.05 vs. baseline Grade 0–1 TR worsened.

**Table 2:** Baseline echocardiographic characteristics of the patient population

	Overall study population (*n* = 852)	Baseline Grade 0–1 TR unchanged (*n* = 583)	Baseline Grade 2–4 TR improved (*n* = 75)	Baseline Grade 0–1 TR worsened (*n* = 85)	Baseline Grade 2–4 TR unchanged (*n* = 109)	*P*-value
LVEDV (mL)	199 (±71)	203 (±71)	201 (±73)	200 (±77)	181 (±64)^*^	0.025
LVESV (mL)	146 (±61)	148 (±61)	151 (±65)	148 (±67)	134 (±58)	0.153
LVEF (%)	27.8 (±8.1)	28.1 (±7.7)	26.1 (±7.9)	27.3 (±8.5)	27.1 (±9.3)	0.205
LAVi (mL/m^2^)	43 (±19)	40 (±17)	46 (±19)	43 (±18)	57 (±24)^*,^^†,^^#^	<0.001
Moderate to severe MR (%)	307 (39.3%)	182 (33.0%)	34 (50.7%)^*^	28 (38.9%)	63 (70.0%)^*,^^#^	<0.001
RVEDA (cm^2^)	22.1 (±7.1)	21.2 (±6.3)	24.3 (±8.8)^*^	22.5 (±7.4)	25.4 (±8.0)^*^	<0.001
RVESA (cm^2^)	14.3 (±6.2)	13.3 (±5.5)	16.7 (±7.6)^*^	15.0 (±6.2)^*^	17.5 (±6.9)^*^	<0.001
RVFAC (%)	36.8 (±12.8)	38.7 (±12.5)	32.8 (±13.4)^*^	34.6 (±13.5)^*^	31.9 (±11.9)^*^	<0.001
TAPSE (mm)	14 (±5)	16 (±4)	15 (±5)^*^	15 (±5)^*^	14 (±5)^*^	<0.001
RA area (cm^2^)	18 (14–23)	17 (14–21)	21 (16–26)^*^	18 (15–23)	24 (20–32)^*,^^†,^^#^	<0.001
TR velocity (m/s)	2.6 (±0.6)	2.4 (±0.5)	2.9 (±0.4)^*^	2.6 (±0.5)^†^	2.9 (±0.6)^*,^^#^	<0.001
PASP (mmHg)	35 (±14)	31 (±11)	43(±11)^*^	34 (±12)^†^	46 (±16)^*,^^#^	<0.001

EDA, end-diastolic area; EDV, end-diastolic volume; EF, ejection fraction; ESA, end-systolic area; ESV, end-systolic volume; FAC, fractional area change; FWS, free wall strain; LA, left atrium; LAVi, left atrial volume index; LV, left ventricle; PASP, pulmonary artery systolic pressure; RA, right atrium; TR, tricuspid regurgitation; RV, right ventricle; TAPSE, tricuspid annular plane systolic excursion.

* *P* < 0.05 vs. baseline Grade 0–1 TR unchanged.

† *P* < 0.05 vs. baseline Grade 2–4 TR improved.

# *P* < 0.05 vs. baseline Grade 0–1 TR worsened.

No or mild TR was noted in 668 (78%) patients at baseline, whereas 184 (22%) had moderate to severe TR. Of the 668 patients with no or mild TR at baseline, the TR grade remained unchanged at 6-month follow-up in 583 (87%) patients, whereas TR progressed to moderate or severe TR in 85 (13%) patients. Of the 184 patients with moderate to severe TR at baseline, TR improved to no or mild TR in 75 (41%) patients, and remained unchanged in 109 (59%) patients at 6-month follow-up. Of the 219 patients who had an upgrade procedure, 85 patients (39%) had two leads crossing the tricuspid valve after the procedure. Of these 85 patients, 50 patients (59%) had no or mild TR at baseline of whom 10 (20%) showed significant TR worsening at 6-month follow-up. In contrast, of the 35 (41%) patients with moderate-severe TR, 12 patients (34%) showed significant improvement at 6-month follow-up.

In per group analysis, patients with no or mild TR at baseline that progressed at 6-month follow-up had more severe symptoms (defined as NYHA functional class III–IV), more impaired renal function, larger RV end-systolic area and more impaired RV systolic function compared to patients with no or mild TR at baseline who had no progression at 6-month follow-up. Those with moderate to severe TR at baseline with improvement after 6 months were younger, more often had sinus rhythm, had smaller left atrial volumes and smaller right atrial areas compared with individuals with moderate to severe TR at baseline who did not improve after 6 months of CRT.

### Changes in clinical and echocardiographic characteristics at 6-month follow-up

**Figure 1:**
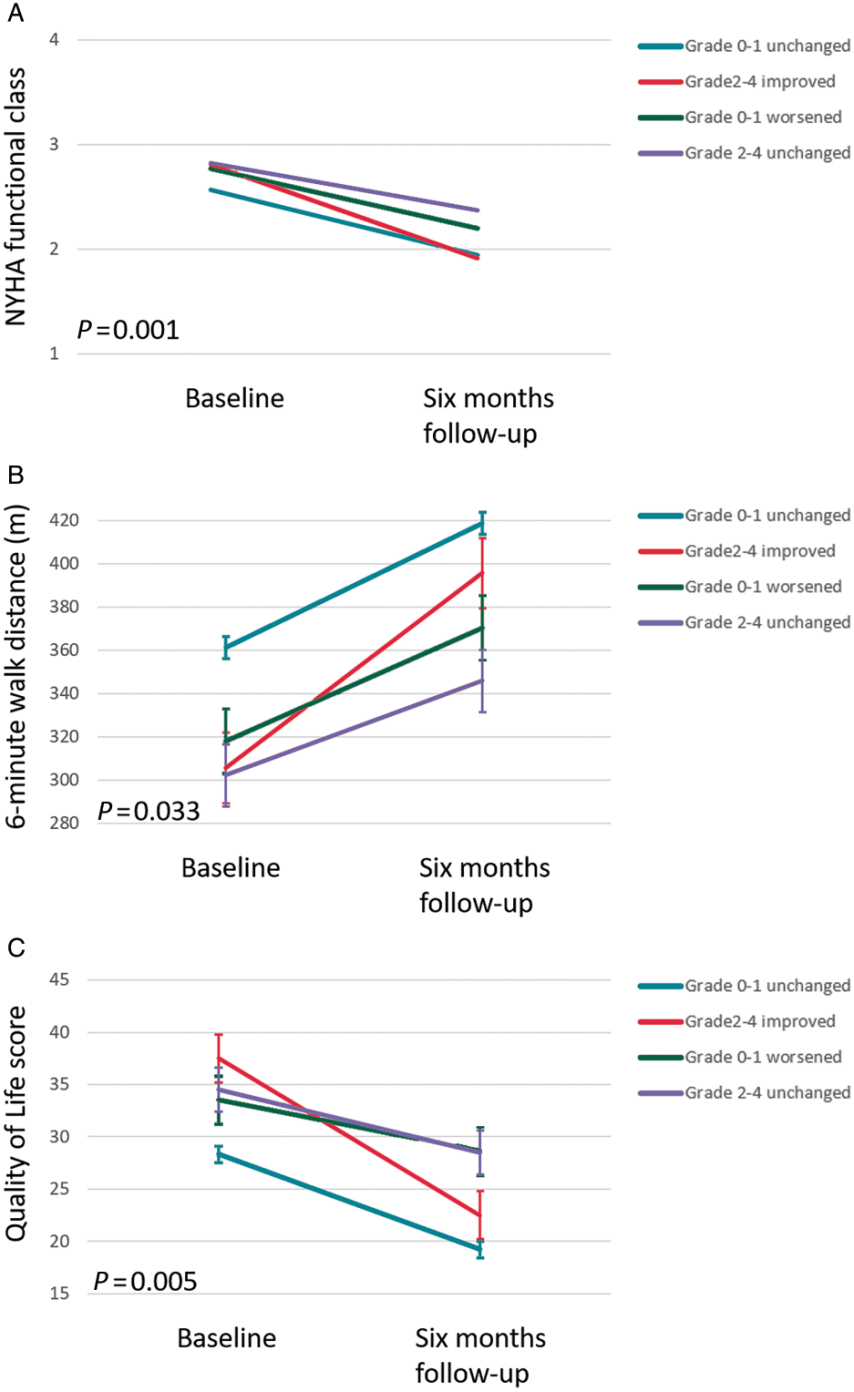
Changes in New York Heart Association functional class (*A*), 6-min walk distance (*B*), and Quality of Life score (*C*), according to different patterns of TR development after 6 months of CRT. Vertical bars represent standard error of the mean. NYHA, New York Heart Association.

**Figure 2:**
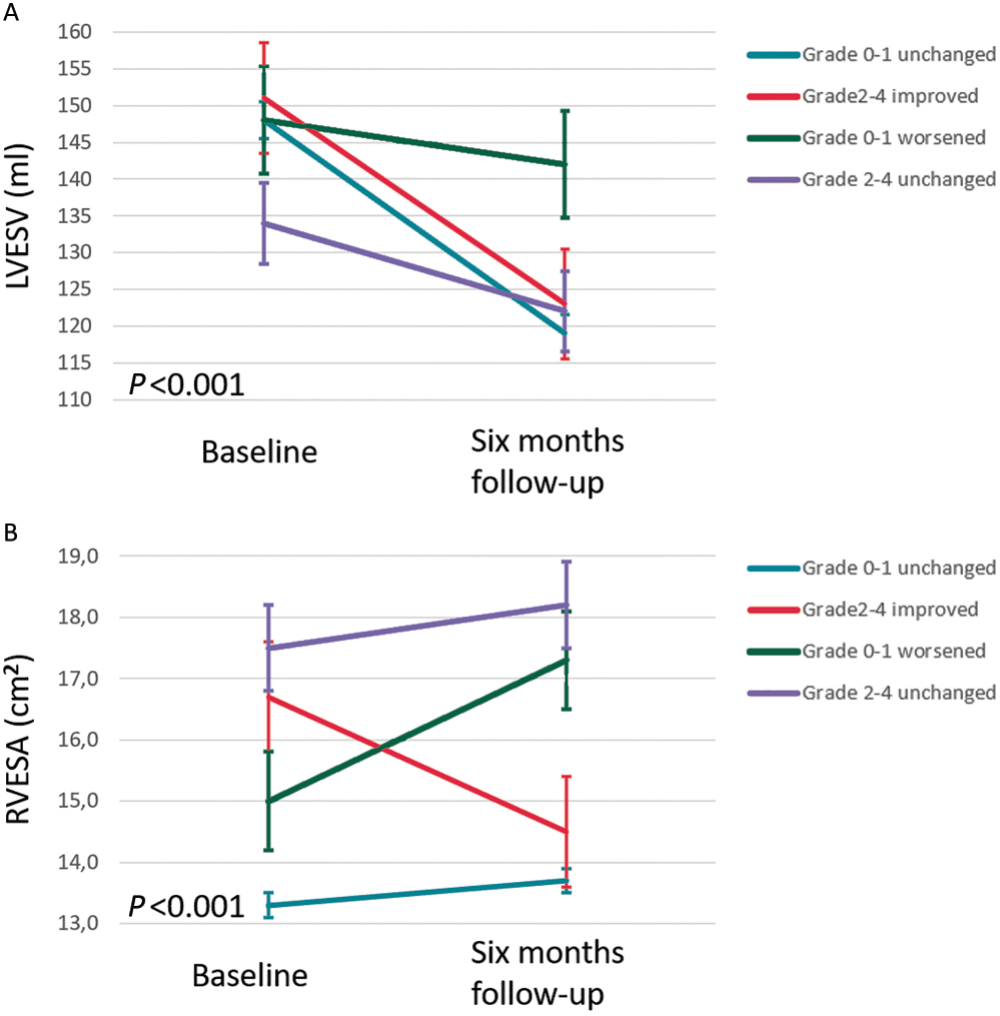
Changes in left ventricular end-systolic volume (*A*) and right ventricular end-systolic area (*B*) according to different patterns of TR development after 6 months of CRT. Vertical bars represent standard error of the mean. LVESV, left ventricular end-systolic volume; RVESA, right ventricular end-systolic area.

The between-group differences of changes in clinical and echocardiographic parameters at 6-month follow-up are summarized in *Table 3*. Changes in clinical and echocardiographic parameters at 6-month follow-up for each TR group separately are shown in Supplementary material online, *Table S1*. Overall, there was an improvement in NYHA functional class (defined as ≥1 class improvement in NYHA from baseline to 6 months), quality of life and 6-min walk test across all TR groups. However, patients with moderate to severe TR at baseline who improved at 6-month follow-up had significantly larger improvement in 6-minute walk distance and quality of life score, compared to patients with moderate to severe TR at baseline which did not improve at 6 months. Changes in NYHA functional class, quality of life and 6-min walk distance at 6 months are shown in *Figure 1*, while changes in LV end-systolic volume and RV end-systolic area are shown in *Figure 2*. There was a greater reduction in LV end-systolic volume and less increase in RV end-systolic area in patients with baseline none to mild TR without deterioration, when compared to patients with baseline none to mild TR who deteriorated at 6-month follow-up.

**Table 3:** Between groups differences of changes in clinical and echocardiographic characteristics of the TR evolution groups

	Overall study population (*n* = 852)	Baseline Grade 0–1 TR unchanged (*n* = 583)	Baseline Grade 2–4 TR improved (*n* = 75)	Baseline Grade 0–1 TR worsened (*n* = 85)	Baseline Grade 2–4 TR unchanged (*n* = 109)	*P*-value
≥1 class improvement in NYHA class (%)	472 (55.4%)	325 (55.7%)	50 (66.7%)	44 (51.8%)	53 (48.6%)	0.095
Absolute change in 6MWT (m)	57.9 (±87.5)	57.4 (±82.3)	90.0 (±90.4)	52.4 (±99.5)	43.6 (±100.9)^†^	0.033
Absolute change in QOL score	−8.8 (±17.1)	−9.0 (±16.6)	−15.0 (±15.9)	−5.0 (±19.1)^†^	−6.0 (±18.5)^†^	0.005
Relative change in RVEDA (%)	5.8 (±24.4)	5.2 (±23.2)	−2.1 (±29.5)	15.7 (±25.6)^*,^^†^	7.6 (±23.6)	<0.001
Relative change in RVESA (%)	9.3 (±31.8)	9.7 (±30.4)	−6.0 (±33.0)^*^	23.3 (±39.2)^*,^^†^	8.1 (±27.7)^†,^^#^	<0.001
Absolute change in FAC (%)	−0.8 (±12.7)	−1.4 (±12.1)	3.6 (±14.3)^*^	−2.3 (±14.9)^†^	0.26 (±12.5)	0.010
Relative change in LVEDV (%)	−8.2 (±21.2)	−10.7 (±20.0)	−5.5 (±24.9)	1.5 (±22.9)^*,^^†^	−2.2 (±20.3)^*^	<0.001
Relative change in LVESV (%)	−15.0 (±24.7)	−18.2 (±22.7)	−16.0 (±27.2)	−1.1 (±30.9)^*,^^†^	−7.8 (±22.5)	<0.001
Absolute change in LVEF (%)	6.1 (±8.9)	6.7 (±8.9)	6.7 (±9.0)	3.2 (±9.8)^*^	4.7 (±8.0)^*^	0.003

EDA, end-diastolic area; EDV, end-diastolic volume; EF, ejection fraction; ESA, end-systolic area; ESV, end-systolic volume; FAC, fractional area change; TR, tricuspid regurgitation; LV, left ventricular; MWT, minute walking test; QoL, quality of life; RV, right ventricular.

* *P* < 0.05 vs. baseline Grade 0–1 TR unchanged.

† *P* < 0.05 vs. baseline Grade 2–4 TR improved.

# *P* < 0.05 vs. baseline Grade 0–1 TR worsened.

### Tricuspid regurgitation evolution and relation with outcomes

**Figure 3:**
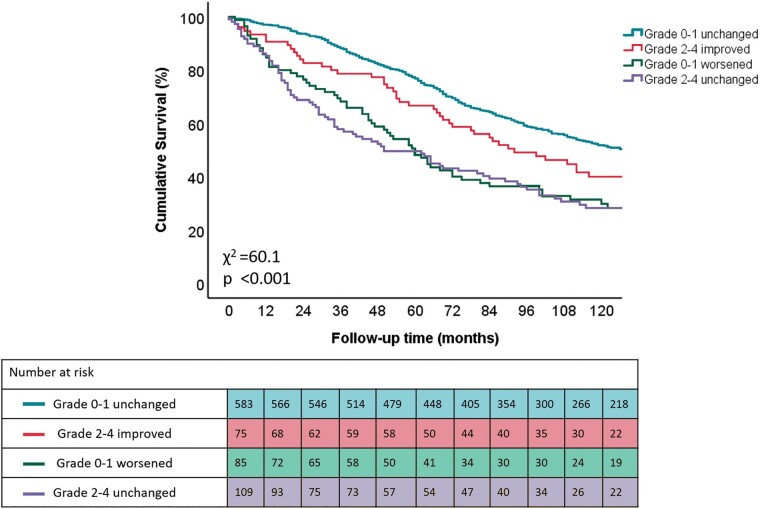
Kaplan–Meier curves for time to cumulative survival, according to different patterns of TR evolution after 6 months of CRT. TR, tricuspid regurgitation

After a median follow-up of 92 (50–137) months, 494 (58%) patients died. The cumulative 1-, 3-, and 5-year survival rates were 94%, 82%, and 70%, respectively. Patients with moderate to severe TR without improvement at 6 months and those with none or mild TR who progressed to moderate or severe TR at 6 months, had the highest all-cause mortality rates during long-term follow-up (*Figure 3*). On multivariable Cox regression analysis, none or mild TR with worsening TR severity (HR: 1.745; 95% CI: 1.287–2.366; *P* < 0.001) and moderate to severe TR without improvement at 6 months (HR: 1.572; 95% CI: 1.198–2.063; *P* = 0.001) were independently associated with higher all-cause mortality, whereas moderate to severe TR with improvement at 6 months (HR: 1.153; 95% CI: 0.814–1.633; *P* = 0.424) was not (*Table 4*). Importantly, this association was independent of CRT-induced RV remodelling.

**Table 4:** Uni- and multivariable Cox regression analyses to assess association between the TR evolution groups and all-cause mortality

	All-cause mortality	
	HR (95% CI)	*P*-value
	Univariable analysis	
Baseline Grade 0–1 TR unchanged	Reference group	
Baseline Grade 2–4 TR improved	1.330 (0.975–1.815)	0.071
Baseline Grade 0–1 TR worsened	2.126 (1.628–2.777)	<0.001
Baseline Grade 2–4 TR unchanged	2.157 (1.687–2.757)	<0.001
	Multivariable analysis^a^	
Baseline Grade 0–1 TR unchanged	Reference group	
Baseline Grade 2–4 TR improved	1.153 (0.814–1.633)	0.424
Baseline Grade 0–1 TR worsened	1.745 (1.287–2.366)	<0.001
Baseline Grade 2–4 TR unchanged	1.572 (1.198–2.063)	0.001

TR = tricuspid regurgitation.

a Adjusted for age, gender, arterial hypertension, diabetes mellitus, hyperlipidaemia, body mass index, ischaemic aetiology of heart failure, atrial fibrillation, New York Heart Association functional class III–IV, estimated glomerular filtration rate, left ventricular end-systolic volume at 6 months, left ventricular ejection fraction at 6 months and relative change in RV ESA at 6 months.

## Discussion

The main findings of the current study can be summarized as follows: (i) moderate or severe TR at baseline is frequently observed in CRT recipients, (ii) moderate or severe TR improved significantly after initiation of CRT in 41% of patients, (iii) patients with moderate or severe TR that improved after CRT implantation had better functional outcomes than those that had no improvement after CRT implantation, and (iv) moderate or severe TR at baseline which remained unchanged after CRT implantation was independently associated with higher long-term mortality. This association was independent of CRT-induced RV remodelling.

### Tricuspid regurgitation in cardiac resynchronization therapy candidates

Significant TR is frequently observed in patients with HF and reduced LVEF, with a prevalence up to 23%.^1^ Most of these patients will have functional TR, occurring from annular dilatation and tethering of the tricuspid valve leaflets due to RV enlargement, which is most often secondary to left-sided (myocardial or valvular) heart disease. Atrial fibrillation, especially if chronic, can also cause or contribute to functional TR by means of tricuspid annular dilatation.^14^ Due to the expanding use of cardiac implantable electronic devices in HF, some individuals may have primary TR, where the device lead interferes with the normal coaptation of the tricuspid valve leaflets.^2^ Significant TR, either primary or secondary, subsequently leads to RA and/or RV enlargement, which induces further tethering and annular dilatation of the tricuspid valve, thereby creating a ‘vicious circle’ of increasing RA/RV enlargement and TR severity.

Significant TR in HF patients is associated with poor long-term prognosis. In a study of 13 026 patients with HF and reduced LVEF, a greater degree of functional TR was associated with considerably worse survival, with a reported 5-year mortality of 55% for moderate TR and 66% for severe TR.^1^

### Effects of cardiac resynchronization therapy on tricuspid regurgitation severity

Functional TR is not primarily a valvular disease, but rather related to RV dilatation/dysfunction, with consequent annular dilatation and leaflet tethering, resulting in leaflet malcoaptation. Therapeutic strategies should therefore aim not only at restoring leaflet coaptation at the level of the valve leaflets, but also at attenuation or reversal of RV dilatation. Although various studies have documented that CRT reduces functional mitral regurgitation by inducing LV reverse remodelling,^15^ the impact of CRT on TR severity has not been evaluated. Cardiac resynchronization therapy has clearly shown the ability to reduce RV dimensions and improve RV function,^8^ and it can therefore be expected to improve TR severity by various mechanisms, including: (i) RV reverse remodelling and reduction of papillary muscle tethering, (ii) reduction of RA size and tricuspid annular area by a decrease in atrial tachyarrhythmia burden,^16^ and (iii) a reduction in RV preload, thereby increasing tricuspid valve closing forces.^8^ Right ventricular reverse remodelling after CRT is likely multifactorial in origin, representing the combined effect of resynchronization and a reduction in pulmonary artery pressure.^8^ Although the effects of CRT on RV papillary muscle dyssynchrony are unknown, an improvement in RV synchrony and contraction efficiency has been documented in CRT recipients with complete right bundle branch block.^17^

In this study, patients with moderate or severe TR at baseline who improved at 6 months after CRT, demonstrated the most pronounced RV reverse remodelling, supporting the hypothesis that CRT-induced RV remodelling underlies most of the TR improvement witnessed after CRT. Conversely, patients with no or mild TR at baseline who progressed to moderate or severe TR at 6-month follow-up, showed more RV adverse remodelling (defined as a relative change in RV end-systolic area).

### Impact of changes in tricuspid regurgitation severity after cardiac resynchronization therapy on long-term prognosis

In the current analysis, TR response to CRT during the first 6 months was associated with increased long-term survival. Moderate to severe TR at baseline which remained unchanged at 6 months after CRT implantation, as well as no or mild TR at baseline which progressed to moderate or severe TR at 6-month follow-up, were independently associated with higher all-cause mortality. The fact that a reduction in TR severity was an independent predictor of survival after adjusting for RV reverse remodelling, suggests that TR reduction is not merely a reflection of the volumetric response of the RV, but that it contributes uniquely to long-term prognosis.

Several mechanisms could explain the beneficial impact of a reduction in TR severity on outcomes after CRT. A significant reduction in TR severity can lead to an increase in RV cardiac output, thereby improving LV preload, LV cardiac output and systemic organ perfusion. A reduction in TR also reduces venous congestion, leading to improved renal function, lower intra-abdominal pressures and less hepatic dysfunction.^18^ In addition, significant TR reduction improves RV volume overload, thereby restoring normal interventricular dependence within the confines of the pericardial space. Since a decrease in TR severity and RV size impact favourably on the LV, this in turn may reduce pulmonary artery pressure and interrupt the vicious circle of pathologic ventricular interaction in HF.

### Therapeutic options for functional tricuspid regurgitation in cardiac resynchronization therapy recipients

In this study, moderate to severe TR at baseline was present in 22% of CRT recipients, while CRT reduced TR severity in a substantial proportion (41%) of these patients. In patients who are eligible for CRT, it might therefore be a good approach to carefully observe TR evolution after device insertion before exploring alternative therapeutic strategies. The high mortality after tricuspid valve surgery^4,^^19^ and modest benefit of surgery in isolated TR,^20^ argue in favour of this approach.

Conversely, the fact that individuals with moderate to severe residual TR at 6-month follow-up had worse outcome, raises the question whether an additional therapeutic strategy might benefit these patients. Transcatheter TR therapies appear promising: the 1-year outcomes of the TriValve registry demonstrated that transcatheter tricuspid valve interventions were associated with better survival and reduced HF rehospitalization, compared to medical therapy alone.^5^ Randomized trials will be required to furnish the necessary evidence regarding the efficacy and timing of transcatheter tricuspid valve intervention in CRT recipients with residual significant TR.

### Study limitations

This was a retrospective, single-centre study. Given the retrospective nature of the study, medical treatment of severe TR, was not standardized. Tricuspid regurgitation severity may be influenced by RV loading conditions, which often vary over time. All-cause mortality was used as the primary endpoint, since no distinction could be made between cardiac and non-cardiac mortality. Moreover, patients who died before 6 months were not included in the analysis, potentially introducing selection bias. Echocardiographic measurements were not performed in an independent echocardiographic laboratory and interobserver differences regarding the assessment of TR severity may have influenced the results. Cardiac magnetic resonance imaging and three-dimensional echocardiography, both of which have the ability to quantify RV size and function more accurately than two-dimensional echocardiography, were not performed. Cardiac implantable device (lead-associated) primary TR remains a challenging diagnosis on two-dimensional echocardiography, and we therefore did not attempt to make a distinction between primary (device-related) and secondary TR.

## Conclusion

Moderate or severe TR improved to no or mild TR after 6 months of CRT in 41% of patients, highlighting the potential benefit of CRT in HF patients with significant TR at baseline. Significant TR at baseline which remained unchanged at 6 months after CRT implantation was independently associated with increased long-term mortality.

## Supplementary material

Supplementary material is available at *Europace* online.

## Funding

J.S. received funding from the European Society of Cardiology (ESC Training Grant App000064741).

## Supplementary Material

euac034_Supplementary_Data

## Data Availability

The data underlying this article will be shared on reasonable request to the corresponding author.
