# Supplementary material for: Tricuspid regurgitation after cardiac resynchronization therapy: evolution and prognostic significance
Source: Europace. 2022 Mar 28;24(8):1291–9. doi: 10.1093/europace/euac034 (PMC9435636; doi:10.1093/europace/euac034)
Supplement: euac034_Supplementary_Data [file euac034_supplementary_data.docx]

**Table S1 – Changes in clinical and echocardiographic characteristics according to TR evolution groups**

|  | **Baseline grade 0-1 TR unchanged**  **(n=583)** | | | **Baseline grade 2-4 TR improved**  **(n=75)** | | | **Baseline grade 0-1 TR worsened**  **(n=85)** | | | **Baseline grade 2-4 TR unchanged (n=109)** | | |
| --- | --- | --- | --- | --- | --- | --- | --- | --- | --- | --- | --- | --- |
|  | Baseline | 6 months | p- value | Baseline | 6 months | p- value | Baseline | 6 months | p-value | Baseline | 6 months | p- value |
| **6 MWT (m)** | 361 (±111) | 419 (±111) | <0.001 | 306 (±125) | 396 (±125) | <0.001 | 318 (±110) | 370 (±113) | <0.001 | 302 (±127) | 346 (±133) | 0.001 |
| **QoL score** | 28.3 (±18.2) | 19.2 (±17.1) | <0.001 | 37.5 (±18.5) | 22.5 (±17.7) | <0.001 | 33.5 (±20.0) | 28.6 (±22.9) | 0.041 | 34.5 (±20.4) | 28.5 (±21.7) | 0.004 |
| **RVEDA, cm^2^** | 21.2 (±6.3) | 22.0 (±10.7) | 0.051 | 24.3 (±8.8) | 22.8 (±8.1) | 0.043 | 22.5 (±7.4) | 25.1 (±7.2) | <0.001 | 25.4 (±8.0) | 26.5 (±8.0) | 0.040 |
| **RVESA, cm^2^** | 13.3 (±5.5) | 13.7 (±4.9) | 0.005 | 16.7 (±7.6) | 14.5 (±5.9) | 0.001 | 15.0 (±6.2) | 17.3 (±6.8) | <0.001 | 17.5 (±6.9) | 18.2 (±7.0) | 0.118 |
| **RVFAC, %** | 38.7 (±12.5) | 37.4 (±11.3) | 0.011 | 32.8 (±13.4) | 36.5 (±14.2) | 0.038 | 34.6 (±13.5) | 32.2 (±12.3) | 0.193 | 31.9 (±11.9) | 32.2 (±11.7) | 0.827 |
| **LVEDV, ml** | 203 (±71) | 179 (±69) | <0.001 | 201 (±73) | 178 (±69) | 0.001 | 200 (±77) | 198 (±76) | 0.748 | 181 (±64) | 175 (±71) | 0.130 |
| **LVESV, ml** | 148 (±61) | 119 (±57) | <0.001 | 151 (±65) | 123 (±58) | <0.001 | 148 (±67) | 142 (±67) | 0.148 | 134 (±58) | 122 (±58) | <0.001 |
| **LVEF, %** | 28.1 (±7.7) | 34.8 (±9.7) | <0.001 | 26.1 (±7.9) | 32.8 (±10.0) | <0.001 | 27.3 (±8.5) | 30.5 (±9.5) | 0.003 | 27.1 (±9.3) | 31.8 (±9.2) | <0.001 |

EDA = end-diastolic area; EDV = end-diastolic volume; EF = ejection fraction; ESA = end-systolic area; ESV = end-systolic volume; FAC = fractional area change; TR = tricuspid regurgitation; LV = left ventricular; MWT = minute walking test; QoL = quality of life; RV = right ventricular.

**
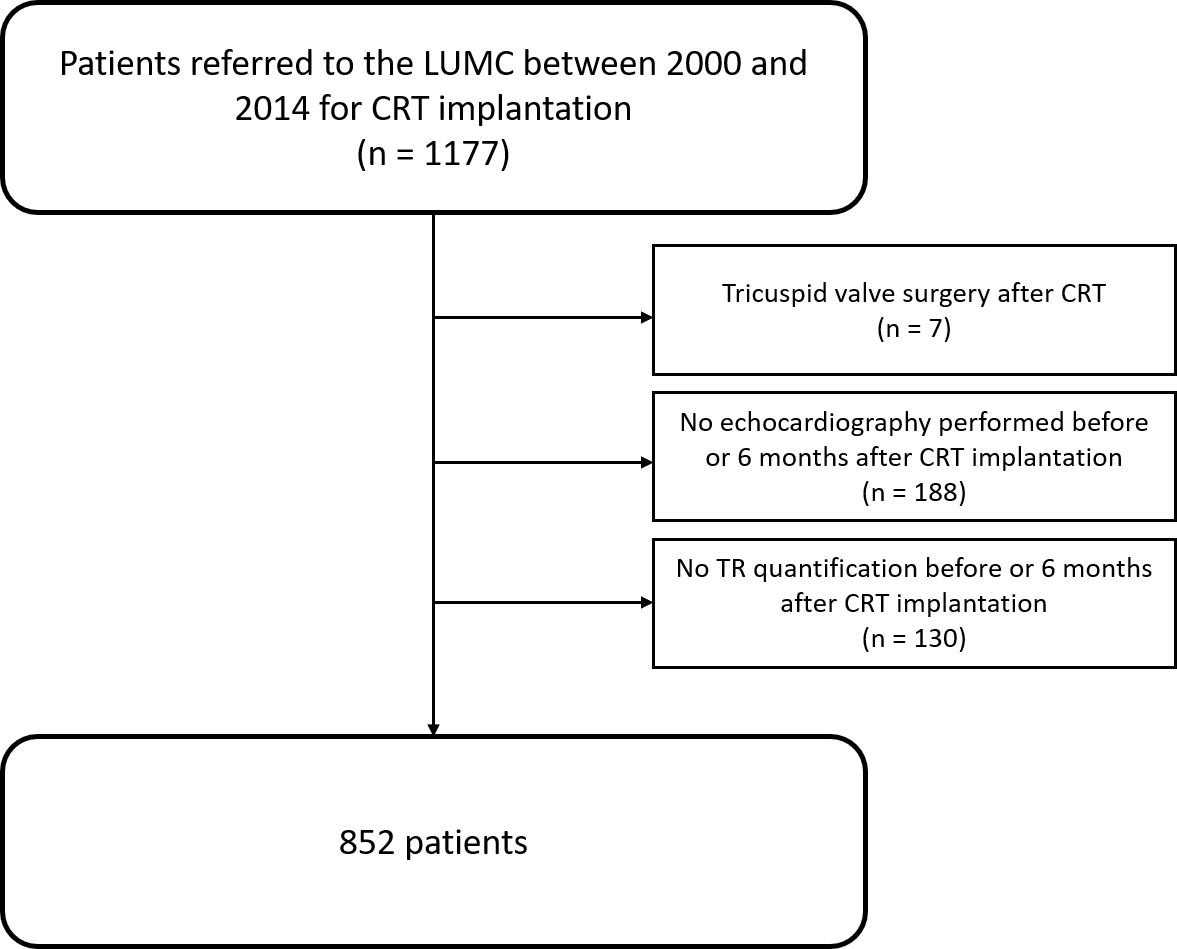
Figure S1 – Flow chart**

CRT = cardiac resynchronization therapy; LUMC = Leiden University Medical Center; TR = tricuspid regurgitation.
